# Lungtech, a phase II EORTC trial of SBRT for centrally located lung tumours – a clinical physics perspective

**DOI:** 10.1186/s13014-015-0567-5

**Published:** 2016-01-20

**Authors:** Marie Lambrecht, Christos Melidis, Jan-Jakob Sonke, Sonja Adebahr, Ronald Boellaard, Marcel Verheij, Matthias Guckenberger, Ursula Nestle, Coen Hurkmans

**Affiliations:** Department of Radiation Oncology, Catharina Hospital, Eindhoven, The Netherlands; EORTC Headquarters, Brussels, Belgium; Department of Radiation Oncology, The Netherlands Cancer Institute, Amsterdam, The Netherlands; Department of Radiation Oncology, University Medical Center, Freiburg, Germany; German Cancer Consortium (DKTK), partner site Freiburg, Germany; Department of Nuclear Medicine VUmc, Amsterdam, The Netherlands; Department of Radiation Oncology, University of Zurich, Zurich, Switzerland

**Keywords:** SBRT, Lung cancer, Image guidance, Radiation therapy quality assurance, Quantitative PET-CT, Phase II trial, EORTC

## Abstract

**Background:**

The EORTC has launched a phase II trial to assess safety and efficacy of SBRT for centrally located NSCLC: The EORTC 22113-08113—Lungtech trial. Due to neighbouring critical structures, these tumours remain challenging to treat. To guarantee accordance to protocol and treatment safety, an RTQA procedure has been implemented within the frame of the EORTC RTQA levels. These levels are here expanded to include innovative tools beyond protocol compliance verification: the actual dose delivered to each patient will be estimated and linked to trial outcomes to enable better understanding of dose related response and toxicity.

**Method:**

For trial participation, institutions must provide a completed facility questionnaire and beam output audit results. To insure ability to comply with protocol specifications a benchmark case is sent to all centres. After approval, institutions are allowed to recruit patients. Nonetheless, each treatment plan will be prospectively reviewed insuring trial compliance consistency over time. As new features, patient’s CBCT images and applied positioning corrections will be saved for dose recalculation on patient’s daily geometry. To assess RTQA along the treatment chain, institutions will be visited once during the time of the trial. Over the course of this visit, end-to-end tests will be performed using the 008ACIRS-breathing platform with two separate bodies. The first body carries EBT3 films and an ionization chamber. The other body newly developed for PET- CT evaluation is fillable with a solution of high activity. 3D or 4D PET-CT and 4D-CT scanning techniques will be evaluated to assess the impact of motion artefacts on target volume accuracy. Finally, a dosimetric evaluation in static and dynamic conditions will be performed.

**Discussion:**

Previous data on mediastinal toxicity are scarce and source of cautiousness for setting-up SBRT treatments for centrally located NSCLC. Thanks to the combination of documented patient related outcomes and CBCT based dose recalculation we expect to provide improved models for dose response and dose related toxicity.

**Conclusion:**

We have developed a comprehensive RTQA model for trials involving modern radiotherapy. These procedures could also serve as examples of extended RTQA for future radiotherapy trials involving quantitative use of PET and tumour motion.

## Background

SBRT of early stage non-small cell lung cancer (NSCLC) has demonstrated high local control rates approximating 90 % and overall survival rates competing with those after surgical resection for patients refusing surgery [1, 2]. While for peripheral pulmonary lesions, mainly surrounded by lung tissue, SBRT with hypo-fractionation and high dose per fraction can be very well tolerated with low risk of treatment-related toxicity, centrally located tumours remain challenging to treat. Several studies have reported unacceptable severe life threatening toxicity [3, 4] due to lesion proximity to mediastinal serial organs. However, thanks to the recent advancements in image guidance and beam delivery different risk adapted SBRT approaches for patients with centrally located NSCLC have been reported to reveal promising local control with modest toxicity [5, 6] thus demonstrating the feasibility of such treatments. Nonetheless, the inhomogeneity among treatment parameters and associated results worldwide [7] has raised a need for prospective large-scale multi-centre studies to investigate the safety and the efficacy of image guided SBRT of centrally located lesions. In response, the EORTC has recently launched a European single arm phase II trial for patients with early stage, centrally located, inoperable NSCLC: The EORTC 22113–08113 Lungtech trial. The primary endpoint of the study is freedom from local progression at three years judged on serial CT scans and in case of suspicion of recurrence additional ^18^FDG PET-CT and if possible biopsy. In addition toxicity is scored according to the Commun Terminology Criteria for Adverse Events (CTCAE. V4) and classified as acute if occurring within 90 days from treatment and as late after 90 days.

The trial presently includes 23 institutions from seven European countries. Anticipating the possible broad type of techniques and installations resulting from the international multi-centre setting, a comprehensive radiation therapy quality assurance (RTQA) procedure has been developed. This procedure aims to assess differences in treatment between institutions, which may induce variations affecting trial outcome [8] and limiting generalisation of the results. Thus, with this comprehensive RTQA program we aim to generate reliable dose–effect and dose-toxicity data, which could be the basis for, improved NTCP and TCP models. We hereby present the RTQA procedures incorporated in this trial. These procedures could also serve as an example of extended RTQA for future radiotherapy trials involving - as in this present trial - tumour motion and a quantitative use of PET data.

## Method

### Protocol and RTQA guidelines

Trial protocols and guidelines may be seen as a first level of quality assurance as they define the main objectives and boundaries of a trial [9].

Since 2006, RTQA requirements for sites participating in EORTC trials have been sorted into five different levels ranging from the minimum procedures, which are required to all trials to trial-specific credentialing implemented depending on the complexity of the technologies involved and the trial aims [10]. As trials become increasingly internationally oriented, harmonisation of RTQA aspects expands as well. From individual to national and finally worldwide RTQA, procedures have been standardized into 10 procedures [11]: facility questionnaire, beam output audit, benchmark case, dummy run, complex treatment dosimetry check, virtual phantom, individual case review (ICR), review of patients’ treatment records, protocol compliance, and dosimetry site visit. The Lungtech RTQA procedure follows five levels defined by the EORTC, which are compliant with these global harmonisation group documents. Moreover, to easily compare dosimetry across patient datasets from the different institutions, a standardized nomenclature is used for target and OAR delineation. Names are compliant with the convention proposed by Santanam et al. [12].

In the following paragraphs the specific RTQA procedures as being performed in the Lungtech trial are outlined; beyond the current standardized RTQA procedures, new QA procedures for 3D or 4D PET-CT and for collection and evaluation of CBCT data for the purpose of dose reconstruction within this trial are described.

### Lungtech trial specific implementations and EORTC RTQA levels

#### EORTC RTQA level 1

The EORTC RTQA level 1, non trial specific, represents the minimal security level in order to insure state of the art radiotherapy treatments can be used . A facility questionnaire defining the type of treatment devices and treatment planning system used must be filled out and results of a beam output audit have to be provided.

In regard to the Lungtech trial, facility questionnaires have been scanned for trial specific requirements; a modern treatment device equipped with a volumetric image guidance system allowing on-line corrections is mandatory (exception made for tracking and gating devices where 2D image guidance systems are allowed). For target definition a 3D or 4D PET-CT to assess tumour volume and also a CT with 4D-CT option to account for target motion in the planning strategy are required.

Differences between dose calculation algorithms in the various treatment planning systems may reach 30 % in individual cases [13, 14] and have lead in some trials to create algorithm specific prescriptions [15]. These differences are mainly linked to whether the algorithm considers changes in lateral electron transport. As the type B algorithms have demonstrated their superiority and are nowadays widely available, the Lungtech trial has set their use as a requirement to ensure limited difference in dose calculation.

An overview of results from the facility questionnaire presenting the combinations of treatment devices, TPS and target motion management techniques used within the trial is given in Table 1.

**Table 1:** Combinations of treatment planning systems, treatment devices and motion management options present within the institutions in which a site-visit has been performed. For some centres several combinations are possible according to their facility questionnaire and preferred combination will be known during the site visit

Treatment planning system	Treatment device	4D option*
Accuray Multiplan	Accuray Cyberknife	Tracking (1)
Brainlab Iplan	Varian Novalis	Gating (1)
Varian Eclipse	Varian Truebeam	ITV (3)
Varian Eclipse	Varian Clinac	ITV (2)
Elekta Monaco	Elekta Synergy	ITV (1)
Accuray Tomotherapy	Accuray Tomo HD	ITV (1)
Philips Pinnacle	Elekta Synergy	ITV (1)

* number of centres using this combination is given between brackets

#### EORTC RTQA level 2: The benchmark case

The benchmark case procedure involves downloading a set of DICOM images of an example patient with relevant patient medical details. Investigators then create a treatment plan according to the protocol instructionsBenchmark case submissions are graded for their conformity to protocol. Plans within tolerance thresholds are considered as demonstrating sufficient competence for trial participation while those with unacceptable variations are not and need to be redone. Radiation oncologists and medical physicists reviewers are blinded to the institutions. Reviews are conducted using VodcaRT™ software integrated in the EORTC RTQA platform [16].

The lungtech protocol follows the promising results of VUmc [5] that treated central tumours with the same hypofractionated approach of applying 8 fractions of 7.5 Gy delivered in an overall time of 2.5 weeks.

The maximum dose inside the PTV should be between 110 % and 130 % of the prescription dose and 95 % of the PTV should at least receive 60 Gy. 99 % of the PTV should receive 90 % prescription dose. In case OAR dose constraints are exceeded two options are considered as acceptable variations: reduction of the prescription dose to 8 times 7 Gy or partial PTV underdosage (Table 2).

**Table 2:** Trial dose specification

	Ideal solution 8 × 7.5 Gy		Reduced prescription to meet OARs constraints 8 × 7 Gy		Underdosed PTV to meet OARs constraints 8 × 7.5 Gy	
Volume	99 %	95 %	99 %	95 %	99 %	95 %
PTV	≥54 Gy (90 %)	≥60 Gy (100 %)	≥50.4 Gy (90 %)	≥56 Gy (100 %)	NA	≥48 Gy (80 %)
CTV	NA		NA		≥54 Gy (90 %) ≥60 Gy (100 %)	
GTV	NA		NA		Acceptable variation	
					66–78Gy (110–130 %)	

The review consists of verification of 1) organs at risk delineation 2) dose specifications (Table 2), please note, the normal tissue constraints used in this trial are discussed in a separate article [17] 3) the use of a motion compensation strategy for target delineation.

Results of the Benchmark case study will be presented elsewhere. Up to date (08/10/2015) twelve centres have submitted their benchmark case study. Eleven were graded satisfactory whereas one centre did not submit a satisfactory case yet .

Site activation is conditioned by the completion of the EORTC first two levels as well as authorization from national ethics commitees. At this cut-off date four centres have been activated.

#### EORTC level 4: Prospective individual case review

Prior to treatment every plan will be reviewed according to the RTQA guidelines. This review is newly to a large extent automated in VodcaRT™. Performed by a trial specific script, it checks the DICOM-RT files for trial specific requirements as: CT slice thickness not exceeding 3 mm, existence of all mandatory structures and DVH constraints. Only then the plan is made available to the reviewers. They review the delineations and dose distribution and provide feedback using a protocol specific online standardised ICR report form.

Unacceptable variations need to be adjusted and central re-evaluation will be performed. When there are no further deviations, treatment plan acceptance confirmation – that is required to start they treatment - will be provided to the site within three working days.

This partial automation of the review should speed-up the reviewer time and shorten the time between plan submission by one institution and approval or rejection from the EORTC thus, allowing a real and complete prospective review.

The EORTC level 3 consists in a rapid, prospective or retrospective review of a limited amount of cases – a lighter version of the level 4 - therefore not presented in this manuscript.

#### Extended EORTC level 4: New implementation - Retrospective dose recalculation

Conebeam CT (CBCT) images before and after each fraction will be collected at the EORTC together with the performed couch shifts. Based on these data, dose will be recalculated retrospectively accounting for the day-to-day set up inaccuracy and patient geometrical deformations. All deformed dose distributions will be transformed back to the original CT acquired for treatment planning and accumulated. Trial specific password protected upload links are available to the institutions.

#### EORTC level 5: Complex Dosimetry check and new implementations

A complex dosimetry check is performed in all the institutions by means of a site visit. All tests are performed in a logical order from image quality used for treatment planning, treatment plans and finally accuracy of plan delivery. The complete procedure allows us to quantify the uncertainty in each step of the treatment chain in each institution. A standardised report will be provided to the institutions containing detailed results and anonymised comparisons with other participating institutes. These observations may be used by the institutions to e.g., further improve their scanning protocols or planning and delivery techniques. No pre-set pass or fail limits were defined for these tests as there was too limited data on clinical useful limits for the required 4D PET accuracy and 4D dose delivery. As no limits were set beforehand, this procedure might be regarded as a quality improvement procedure rather than a quality assurance step.

##### Phantom details

**Fig. 1:**
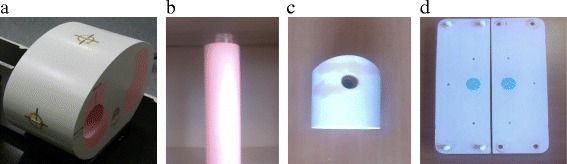
**a** CIRS 008A body - **b** rod simulating a breathing lung - **c** 15 mm diameter sphere ionization chamber insert - **d** 15 mm diameter sphere film insert

For this study the CIRS 008A model (Computerized Imaging Reference Systems, Norfolk, Virginia, USA) will be used. This dynamic thoracic phantom is an anthropomorphic phantom with two lung shaped regions with lung equivalent density material, a water-equivalent mediastinum and a vertebral structure. A lung equivalent density rod inserted in the phantom’s right lung contains a spherical target of water equivalent density simulating a lung lesion. A motion actuator moving the target according to a respiratory signal specified by the user can drive the rod. The rod may be used with different type of dosimetric inserts. In this study a film insert and a set for a 0.04 cm^3^ ionization chamber are used. The micro-chamber inserts are machined to receive the dosimeter at the centre of the target volume. Regarding the film inserts, to allow the placement of the gafchromic EBT3 films in the middle plane of the spheres, the spheres are constructed as two one-half spheres (Fig. 1).

**Fig. 2:**
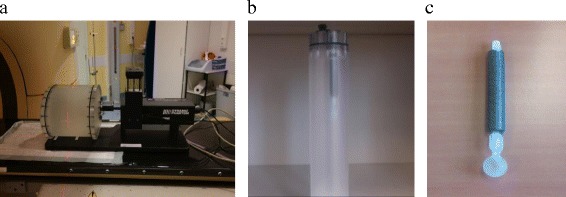
**a** Customized PET CIRS phantom plugged on the breathing platform - **b** PET rod simulating the lung compartment - **c** 15 mm diameter sphere simulating a lung lesion

In addition to this anthropomorphic body a customised PET-CT body has been developed in collaboration with CIRS (Fig. 2). Positioned on the same breathing platform the PET body consists in a fillable thorax body of 18 cm length and a fillable cylinder in which a hollow sphere can be inserted which can be filled with a higher activity-mimicking tumour ^18^FDG uptake. Two sphere diameters of 15 and 25 mm will be used. To simulate the breathing motion, the equation proposed by Lujan et al. [18] is used with n set to 3 (equation 1).

1 $$ s(t)={s}_0+A\cdot { \cos}^{2n}\left(\frac{\pi t}{\tau }+\phi \right) $$

Several studies have reported breathing cycles for lung cancer patients varying between t = 3 and 6 s [19–21]. Both 15 and 25 mm longitudinal peak-to-peak amplitude (A = 7.5 and 12.5 mm) will be tested. Due to phantom mechanical limitations, the combination of a 3 s breathing cycle with 25 mm amplitude is not possible. This combination has therefore been replaced by 25 mm amplitude with a breathing cycle of 4 s (Table 3).

**Table 3:** Combination of breathing parameters

	Test 1: Spheres	Test 2: Spheres	Test 3: Sphere
	15 and 25 mm	15 and 25 mm	15 mm
Breathing Period (s)	6	3	4
Peak to peak Amplitude (mm)	15	15	25

##### PET-CT credentialing

No ^18^FDG fixation threshold has been set in this trial for target contouring, but PET-CT images are used as an informative tool in target delineation. Moreover ^18^FDG PET-CT is planned to monitor treatment response as translational endpoint, including staging comparison between 3D and 4D – as applicable - ^18^FDG PET-CT assessment. Although PET scans are widely available, their use in multicentre clinical trials is challenging. Differences in quantitative values between PET images from different institutions may simply occur due to differences in scanner performance as a result of using different reconstruction settings [22].

Not all centres participating in this trial are EARL accredited http://earl.eanm.org/cms/website.php?id=/en/projects/fdg_pet_ct_accreditation.htm therefore it is of interest to investigate SUV consistency among these institutions.

This RTQA procedure will allow us to pool data from 23 institutions using the same phantom replicating biological fixation rates in respectively mediastinum, lung and tumour compartments and to analyse recovery coefficients using different scanner brands and different reconstruction algorithms and parameters. In addition the procedure evaluates the impact of several breathing motions on SUV values.

The PET phantom body, cylinder and sphere are filled with homogeneous solutions of water and ^18^FDG produced locally. The activity concentrations are set using the institution prescription for an average patient of 70 kg and recalculated for the phantom weight (6 kg) with a lesion-to-background ratio of 10 [23, 24] to simulate lung lesions. After filling the phantom, activity concentrations of each compartment are sampled and checked for absolute measurements.

PET-CT images are reconstructed using institution settings and the parameters described in Table 4 are saved. Recovery coefficients are calculated for each sphere size and each motion.

**Table 4:** PET stored parameters

PET	Static	15 mm/3 s	15 mm/6 s	25 mm/4 s
Type	3D	4D:10.phases	4D:10 phases	4D:10 phases
Acquisition time	12:30	12:48	13:04	13:20
TOF	YES			
Binning type	X	Phase binning		
Number of bed position	2	1		
Overlap bed	90 mm			
Time per bed position	2 min	10 min		
Data corrected for:	decay corrected : aquisition start time			
	attenuation corrected			
	scatter corrected			
	dead time corrected			
	randoms corrected			
	non-uniform radial sampling corrected			
	detector normalizationu			
Type of reconstruction algorithm	BLOB-OS-TF			
Energy window	440–665 KeV			
Slice thickness	4 mm			
Image matrix	144 × 144			
FOV	903 mm			

Several recovery coefficients are derived using different uptake parameters: maximum, average and volume recovery coefficients.

Maximum recovery coefficients (RCmax) are calculated using the maximum pixel value compared to the absolute activity concentration measured on the samples.

2 $$ RCmax = \frac{Maximum\  pixel\  value\  inside\  the\  sphere}{Measured\  activity\  concentration} $$

The average recovery coefficients (RC50) are calculated for a sphere size using 50 % of the maximum pixel value on the static PET-CT as threshold to draw on each phase an isocontour VOI50. The average pixel value inside this VOI50 is then as previously compared to the measured activity.

3 $$ RC50=\frac{Average\  pixel\  value\  inside\  the\ VOI50}{Measured\  activity\  concentration} $$

Finally the VOI50 of each phase and on the average PET-CT are compared to the VOI50 derived from the static image and result in volume recovery coefficients (VRC50).

4 $$ VRC50=\frac{VOI50x}{VOI50\  statique} $$

The same process is repeated with 70 % of the maximum SUV giving respectively the RC70 and the VRC 70.

**Fig. 3:**
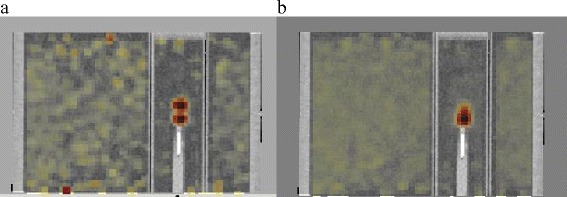
CIRS phantom with the 15 mm sphere size animated with a 15 mm/6 s breathing signal (**a**) Superposition of 10 % and 40 % phases (the most distant sphere positions) PET-CT images - (**b**) Average PET-CT

**Fig. 4:**
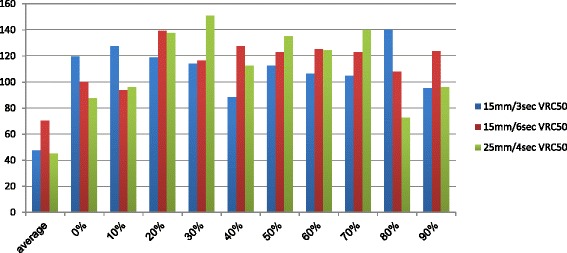
Volume recovery coefficients VRC50 (VRC50 = VOI50/VOI static) expressed in % of VOI static. Single site results aquired on a Gemini TF PET-CT Big bore

**Fig. 5:**
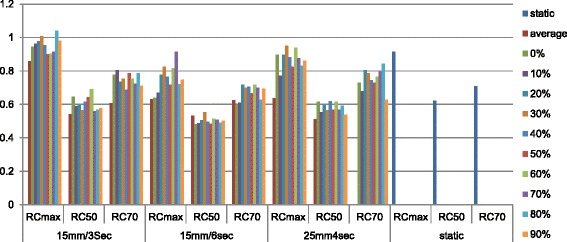
Activity recovery coefficients (RCmax, RC50, RC70) of the 15 mm sphere calculated in static and dynamic conditions (static PET and 4D PET). Single site results aquired on a Gemini TF PET-CT Big bore

Examples of saved parameters and 4D PET-CT calculated recovery coefficients are presented in Table 4 and Figs. 3, 4 and 5.

##### Credentialing of 4D-CT scan techniques

**Fig. 6:**
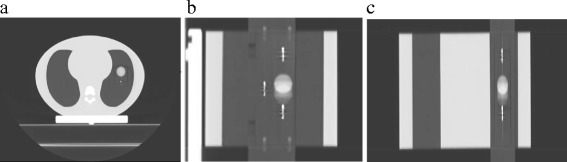
Average 4DCT of the CIRS phantom with the 25 mm sphere animated with a 15 mm/6 s breathing motion **a** transversal **b** sagittal **c** coronal

**Fig. 7:**
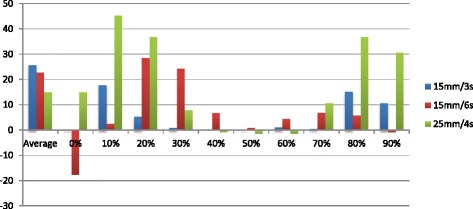
Volume variation induced by the three breathing motions and expressed in per cent of the static volume for the 15 mm sphere. Single site results aquired on a Gemini TF PET-CT Big bore using a phase binning approach

Institutions are required to take motion into account in their planning strategy. They are nonetheless free to use the solution of their choice; ITV, mid-position, tracking or gating. The accuracy of theses strategies all depend on the 4D-CT imaging quality (Fig. 6). We thus developed a test procedure to evaluate the impact of motion on the target volume and motion as determined using the available binned CT data. The same static and dynamic condition as presented in Table 3 are used when scanning the anthropomorphic thorax phantom on the CT scanner used for planning. Knowing the exact volume of the sphere, the HU threshold on the static CT, which results in the true volume, will be used for autocontouring the 4D-CT datasets. The threshold is applied on each of the phases of the 4D-CT thus the volume can be calculated on each phase (Fig. 7). Then the centre of mass of the auto-segmented contour in each phase is used to calculate the motion amplitude captured by computed tomography. This result will be evaluated and compared to the known motion amplitude (Table 5).

**Table 5:** True peak to peak amplitude of the 15 mm diameter sphere versus measured on CT from one of the participating institutions

	LAT		ANT-POST		SUP-INF	
	true	Measured	true	Measured	true	Measured
A:15 mm	0	0.02	0	0.04	1.5	1.4
C:3 s						
A:15 mm	0	0.06	0	0.01	1.5	0.87
C:6 s						
A:25 mm	0	0.07	0	0.07	2.5	2.21
C:4 s						

##### Credentialing of radiotherapy delivery

Treatment plans will be made by the institutions based on the trial protocol dose specifications for the two spheres in static conditions and for the 15 mm diameter sphere for the 15 mm/3 s breathing motion. These plans will be measured twice successively using both EBT3 film and a 0.04 cm3 ion chamber. The static measurements should agree within 3 %/3 mm with the calculated dose based on a gamma analysis using the dose values above 20 % of the prescription dose. The analysis will be performed using the absolute dose, corrected for daily output changes and with the calculated dose as the reference dose.

## Discussion

Several studies have reported a need for prospective multi-center data for centrally located lung tumours [25]. Previous data on mediastinal toxicity are scarce and source of extreme cautiousness for setting-up SBRT treatments for centrally located lung tumours. We hope that the here presented intense RTQA procedure with individual dose recalculation will provide the data to derive robust TCP and NTCP model parameter (e.g., using Lyman-Kutcher-Burman and log-logistic models) [26]. This will aid the safe further spread of these treatments to other clinics. The primary end point of the trial is local control, which will be assessed on CT by evaluating the tumour size . In case of equivocal results an FDG PET-CT will be performed. Huang et. al. have defined a set of criteria to assess recurrences on PET and CT images. They proposed an SUVmax > 5 as threshold for recurrences [27, 28]. This threshold would be valid for PET-CT scanners, which are EARL accredited. However since not all centres within the Lungtech trial are EARL accredited a modified Huang criteria was defined, as “focal FDG accumulation significantly above the mediastina blood pool”. Nonetheless thanks to the quantitative analysis of the RTQA we will have information regarding the variability of the SUV values within the institutions, which may allow us to validate the Huang criteria as response criteria for multi-center studies. The trial will also intensively investigate acute and late toxicities. Toxicities will be rated according to the “common terminology criteria for adverse events” v.4 and these data will be correlated to the recalculated dose for each OAR. We also will evaluate toxicity in relation to institution specific techniques and equipment [29].

Other already closed or on-going SBRT lung cancer trials differ in their requirements and RTQA methodology. As for example, the RTOG 0813 phase I/II SBRT trial for early stage, centrally located, NSCLC in medically inoperable patients aims to determine the maximum tolerated dose. An extensive RTQA procedure has been set to ensure an acceptable safety level, comprising 5 steps as well. However, a dummy run instead of a benchmark case test is used in the RTOG trial (in a dummy run procedure institutions plan accordingly to protocol one patient case of their own). This not only makes comparisons between institutions more difficult, but it can be expected that institutions will send for review one “convenient” patient of theirs and not one chosen by the trial’s RTQA committee. Also, 3D image guidance is not mandatory in that trial, preventing the possibility to recalculate the given dose based on CBCT data.

## Conclusion

We have developed a comprehensive RTQA program for trials involving modern lung radiotherapy including 4D imaging. This program should not only largely prevent non-compliant protocol treatments, but also enable calculation of treatment uncertainty and CBCT based dose recalculation. Thanks to the combination of well documented patient related outcomes and CBCT based dose reconstructions, we expect to provide improved models for dose response and dose related toxicity in the challenging context of centrally located NSCLC treated with SBRT.

The EORTC RTQA program has been implemented for the Lungtech trial. As the new procedures are optimally harmonised to global RTQA trial procedures, incorporating a 4D PET-CT QA phantom and including full retrieval of 3D CBCT data, we believe this program can act as a template for many future trials.
